# Structural equation modeling approach for the analysis of ergonomics risk factors and occupational injuries among building construction workers in Bahir Dar City-Ethiopia

**DOI:** 10.1016/j.heliyon.2024.e32234

**Published:** 2024-05-31

**Authors:** Gebeyaw Tadele Belay, Bereket Haile Woldegiorgis, Yogi Tri Prasetyo

**Affiliations:** a University of Gondar, Institute of Technology, Department of Industrial Engineering, Gondar, Ethiopia; b Bahir Dar University, Bahir Dar Institute of Technology, Department of Industrial Engineering, Bahir Dar, Ethiopia; c International Bachelor Program in Engineering, Yuan Ze University, 135 Yuan-Tung Road, Chung-Li, 32003, Taiwan; d Department of Industrial Engineering and Management, Yuan Ze University, 135 Yuan-Tung Road, Chung-Li, 32003, Taiwan

**Keywords:** Bahir dar city, Building construction workers, Ergonomics risk factors, Occupational injuries, Safety management practice, Structural equation modeling

## Abstract

Ergonomic risk factors are a prominent cause of fatality and severe injuries in building constructions. Hence, this study applies a Structural Equation Modeling (SEM) approach to analyze ergonomics risk factors and occupational injuries among building construction workers in Bahir Dar City, Ethiopia. The results indicate significant relationships between ergonomics risk factors and the prevalence of occupational injuries. This study's findings contribute to the understanding of occupational health and safety in the construction industry, highlighting the need for targeted interventions. A cross-sectional study has been carried out, where data was collected through direct observations and standardized pretested questionnaires. The study recruited 220 participants in the construction industry. The data was analyzed using AMOS to study the direct and indirect effects of the identified variable. SEM has shown that the magnitude of the prevalence of occupational injury was 65.2 %. The results also revealed that the mostly affected body parts were lower arm, lower leg, hand, toe, and knee. Carpenter & roofers, plasterer and daily labors & other helpers were highly injury subjected occupations in respective order. The six leading significant risk factors were, do not tie belt at scaffold, falling stairways & ladders, exposure hazardous substances, tools & machinery, electricity (electric power accidents), repetitive tasks, the layout didn't consider health & safety aspects in the site, and do not provide safety orientation for new workers engaging the job. Employees are mostly affected on their lower body parts which needs more focus to prevent it, especially carpenter & roofers, plasterer and daily labors. Also the findings show that 50 % of respondents agree that the higher priority for safety and health management practices should be given to finishing part of the construction followed by excavation and earth work, masonry, and electrical installations. Therefore, it is recommended that the contractors must focus more on the finishing phase.

## 1 Introduction

### 1.1 Background of the study

**Fig. 1:**
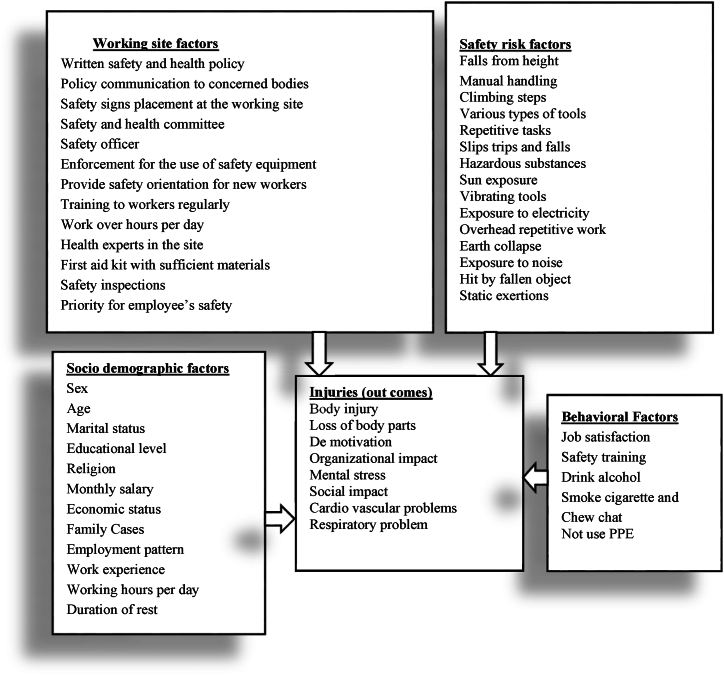
Conceptual framework for causal relations of variables for injuries.

**Fig. 2:**
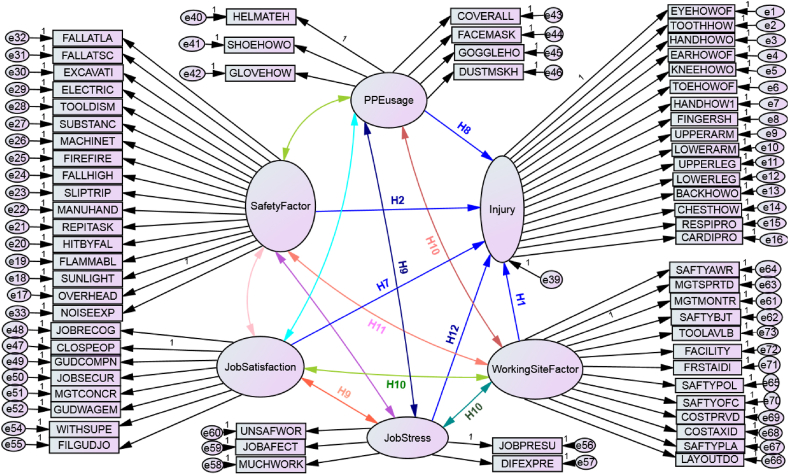
Risk factors and injuries causal relationship model; modeled by SEM-Amos.

Globally, as of International Labor Organization 2023 report in Australia, over 395 million workers are subjected in a non-fatal work injury in 2019. In addition, around 2.93 million workers died as a result of work-related factors, which is an increase in more than 12 per cent compared to 2000. In this report, agriculture, forestry and fishing, construction, and manufacturing sectors are widely recognized as the most hazardous sector. Each year, 200,000 fatal injuries occur in these sectors, representing over 60 percent of all fatal occupational injuries. Constructions, such as roads, buildings, dams, and the bridges has been stated as one of the most injury-prone sectors both in developed and developing countries [1,2]. Some of the major reported causes of injuries the construction industry are awkward postures, lifting heavy materials, manual handling of heavy and irregular-sized loads, frequent bending, twisting, working above the shoulder height, working below the knee level, staying in one position for a long period, repeated climbing and descending, and pushing and pulling of loads [3]. The growth of the construction industry increases unfavorable ergonomics risk factors which include numerous activities, therefore, injury and fatality rates become higher. As per Hoła and Szóstak [4], construction workers are subjected to various work-related injuries and occupational hazards, such as slipping and falling, injured by fallen object, musculoskeletal disorders (MSDs), skin disease, eye injury, breathing system difficulty, cardiovascular problem, hearing and other sensing human body problems. Similarly, Khashaba, El-Helaly, El-Gilany, Motawei, Foda et al. [1] listed the construction injuries as falls (both slipping and from rooftops), machinery (both failure and unguarded), struck by fallen materials, electric problems, dust, welding swags, temperature, and other mechanical accidents. Studies also show that even non-occupational diseases may be worsened by activities in the construction, which sometimes may lead to serious diseases or to death. These fatal accidents do not only cause heavy losses in property and lives, but also has very high personal, social and financial costs [5] (see Table 1, Table 2, Fig. 1, Fig. 2, Fig. 7).

**Table 1:** Test of model fits in Amos.

No	Fit tests	Cut point	Current study	Source
1	*X*^*2*^/df	≤3	1.914	[[45], [46], [47]]
2	Goodness of fit index (GFI). Near to 1 is perfect fit	0 ≤GFI ≤1	0.63	[[45], [46]]
3	Root mean square residual (RMR). Zero is a perfect fit	≤0.08	0.058	[46]
4	Root mean square error approximation (RMSEA)	≤0.1	0.067	[24]
5	Comparative fit index (CFI). Near to 1 is perfect fit	>0.731	0.735	[24]

**Table 2:** Standardized regression weights: on SEM path modeling.

Out come	Effect direction	Risk factors	Direct effect	P-value
Injury	<---	Safety Factor	−0.483	0.001
Injury	<---	Working Site Factor	0.005	0.942
Injury	<---	Job Satisfaction	0.286	0.004
Injury	<---	Job Stress	0.326	0.001
Injury	<---	PPE usage	−0.294	0.005

In Hoła and Szóstak [4], about 53.4 % of the construction related injuries are reported from assemblers, carpenters, concrete workers, steel fixers, road construction workers, bricklayers, and related professions. However, Timofeeva, Ulrikh, and Tsvetkun [6] concluded that electrical and gas welders, followed by carpenters and concrete workers, were the most frequent injury prone activities in the construction. The studies also show that age and experience [4] were some of the factors that affect the frequency of occurrence of accidents. Similarly Kim [3] argued that the three most frequently affected body parts were shoulders, neck, and back.

In the Ethiopian context, there are limited studies reporting the prevalence and distribution of accidents in the construction sector. The results at different parts of the country revealed that the overall distribution of work-related injuries were 38.7 % [7], 34.9 % [2], 46.2 %[8], 34.6 % [9], 41.4 % [10], 76.2 % [11], 39.2 % [12], 32.6 % [38], 66.7 % [13] and 43.9 % [34]. Likewise, lower limbs (73.2 %) and upper limbs (16 %) are the two most commonly injured body parts [2], where the overall annual prevalence rate of 346 injuries per 1000 construction workers were observed out of which 77.7 % of them needed to be hospitalized [9]. Also in Ref. [12] identified falls from the same level (38.5 %), injuries caused by movable or falling objects (23.1 %), and falls from elevation (13.4 %) as the three major classes of injuries, whereas the leading injuries in Ref. [10] were caused by an object (36.9 %), followed by lower back pain by twisting and bend over (35.6 %), falling due to sleep and trip (23.5 %).

In the study of Anna Arlinghaus and Christiani ([35]) have used structural equation modeling (SEM) as a methodology to analyze risk factors for work-related occupational injury with a sample size of 89,366 employed workers by using cross-sectional study. Gender, age, race/ethnicity, occupation, type of pay, and psychological distress were modeled in the SEM. From those variables, long working hours and short sleep duration were high-risk factors. The next risk factors were gender, occupation, and type of pay. These studies showed that SEM methodology was the best approach, because this method can show risk factors and injuries for both direct and indirect causal factors of variables in a complex work system, although the above study's methodology were not analyze the indirect effects. From the total injuries, more than half (68.39 %) were reported by males while the rest reported by females workers. The leading causes of injuries were, fall from ground level (21.3 %) followed by overexertion during lifting (20.6 %), and fall from elevation (16.1 %). Old age, being male, job dissatisfaction, lack of training and working overtime; were the risk factors for having occupational injuries among construction workers. But in Gebremeskel [38] negligence of the workers and lack of awareness were factors significantly associated with occupational injury. Gender, educational status, safety training, personal protective equipment were statically significantly associated. Also Miruri [9] showed the main cause of the injury; injured while they were fight by sharp object 166 (59.7 %) and hit by falling object 47 (16.9 %). In Tran [14] the result showed that, 6 immediate factors that have a direct impact on falls from the heights those were: operational equipment, PPE, employee mental attitude, safety awareness, environmental conditions, and worker's skills. In this study, there were also 3 organizational factors (preplanning, management/supervision, and training), 3 policy factors (design for safety, corporate culture, safety and health management) and the last is regulatory requirements was a unique factor to outside contractor's organizations.

Following ergonomic principles in constructions could reduce project cost overrun and injuries and fatalities through introduction and implementation of safety & health practices (P. Entzel, J. Albers, & L. J. A. E. Welch, [44]; Hess, Weinstein, Welch, & hygiene, [40]). However, for instance even though 90 % of the US construction industries with 6 to 3000 workers have a written safety and health programs, only 30 % of them have in the actual practice [15]. It seems, the management and workers in the construction industry tend to accept the fact that construction work is just a complex and heavy task-oriented sector, accidents and injuries are inevitable [15]. On the other hand, studies show that dissemination, adoption, and implementation of effective external interventions or practical researches can have significant roles in prevention of injuries ([44]). Therefore, fostering principles of ergonomics in all phases of the construction works such as engineering, pre-planning, purchasing, materials handling, job site management, and training of supervisors and workers, the construction industry can take the burden off workers, isolate accidents, and reduce WMSDs (Pamela Entzel et al., [37]).

To prevent these risks of losses; distribution of occupational injuries and occupational risk factors have to be identified and quantified. Furthermore, studies show that the distribution of occupational injury varies from place to place and even from site to site. Therefore, it is necessary to understand the magnitude of the problem at different construction sites and places. Hence, the aim of this study is to assess ergonomics risk factors and the magnitude of occupational injuries among building construction workers. This study may use as baseline information for policymakers to make decisions, to put regulations and also may play a great role for future researchers those who are interested further investigation on construction work-related injuries and the associated factors to incorporate for national and international context, also it uses for employer and employee to design a strategy for preventing both together work-related injury with the risk factors.

Numerous studies have explored ergonomics risk factors in the construction industry. However, there is a lack of research focusing specifically on the interplay of these factors in the Ethiopian context. Recent studies ([[49], [50]] have highlighted the importance of addressing ergonomic risks to reduce occupational injuries.

This study aims to fill the research gap in understanding the specific ergonomics risk factors contributing to these injuries. Also it emphasizes the global relevance and critical need for assessing ergonomics risk factors and occupational injuries in the construction industry. By using SEM, we provide a comprehensive analysis of these risk factors and their interrelationships, offering insights for improved safety management practices. This study also highlights the unique contributions, unlike previous studies; the study employs SEM to analyze the direct and indirect effects of various risk factors on occupational injuries. This methodological approach allows for a comprehensive understanding of the interactions between different variables, setting this study apart from others that may have used simpler statistical methods. Additionally, the study's focus on a specific region with its unique socio-economic and working conditions adds a novel perspective to the existing body of literature on construction safety. This focus adds significant value as it fills a gap in the literature for this geographical area. Moreover the study offers baseline data for policymakers and stakeholders to develop targeted interventions to improve safety standards and reduce injury rates in the construction sector.

## 2 Methods and materials

A cross-sectional and deductive study was carried out. Structural Equation Modeling (SEM) was used which can have different software's, but, in the current study, Analysis of Moment Structure (AMOS) version 23 was specifically used, which essentially applicable for modeling the relations of variables as a researcher aims to do. The six-step process for structural equation modeling method of four steps in Wu [16] and one step from Zhou, Zhe-Hua, and Sun ([43]) were used in the study. To accomplish this study, personal observations in construction sites were done for the research design, a standardized pretested questionnaire was prepared for required data collection [9,10,12]. To gather the information about disabled workers who are leave the site because of being injured by construction-related accidents, and fatal related accidents or deaths, an interview was conducted directly to the administrative staffs and secondary data sources from the record were gathered. The sample size was determined by using the principle of Structural Equation Modeling (SEM). The sample size in this statistical approach is recommended to be not being less than 200. But in case of errors and nonreturnable allowances were considered as, the margin of error 5 %, consider 5 % nonresponse rate and 95 % confidence interval. Then the questionnaire was given to the respondents by using a simple random sampling method. Respondents were clustered by their purposes; accordingly, the desired and accurate data were collected. Stopwatch, EpiData, AMOS and questionnaire tools were used for data collection, analysis and modeling of relational factors.

### 2.1 Questionnaire and sampling

A questionnaire was developed based on previous studies [9,17] and the specifics of the case study. Eleven respondents’ data (5 % of the total sample size) was used for the pre-test. The respondents in this survey were groups of individuals and experts in the building construction. After the pilot survey, wordings, questionnaire contents, layouts, comments, and some ideas were revised.

After the pilot study was completed, a cluster sampling technique was employed to select construction workers based upon their occupation. Participants were selected randomly from ten sites. They were further clustered into eight purposive samples for each construction site; daily laborers, plasterers, carpenters, masons, painters, metal workers/fetters, electricians including welders, and machine operators. The number of samples from each site was determined by using proportional allocation as per the number of population found in each site. The sample size determination was done by using the principle of Structural Equation Modeling (SEM) which should be between 200 [16] and 400 [18,19], and (Harris, [39]). Therefore, the total sample was 220 by assuming 95 % confidence interval, margin of error 5 %, and non-response rate of 5 % (Mersha, [42]).

### 2.2 Data collection procedure

The raw data were collected from 204 respondents across various building construction sites using multiple methods. A detailed questionnaire was one method. Additionally, interviews and survey were conducted with construction administrative staff to gather information on fatal accidents, capturing data on workers who were not present during the survey due to severe disability or death. Illiterate workers were also interviewed. Secondary data were sourced from the Bahir Dar city labor and social affairs administration and the city's development and house construction office. Ergonomic instruments were used to measure risk factors.

The questionnaire was designed for a cross-sectional survey and deductive research, involving hypothesis development and risk factor modeling. Respondents were selected randomly using a lottery method. Out of 220 distributed questionnaires, 204 were completed and returned, slightly exceeding the target of 200 to account for a 5 % margin of error and a 5 % nonresponse rate.

A total of 16 questionnaires were rejected: 8 were returned blank, 7 were incomplete, and 1 was not returned. This resulted in a 92 % response rate. The collected data were entered into EpiData 3.1 software to ensure accurate data recording and to prevent errors. The data were then exported to SPSS V-20 for analysis. For further analysis and modeling of causal relationships, the data in SPSS were exported to AMOS V-23 software.

### 2.3 Conceptual framework

All occupational injuries on construction workers are considered to be as dependent variables. Those variables are directly or indirectly affect the workers, for example, temperature, humidity, fallen objects, sleep, and so on. Also, injuries or endogenous variables like cuts, back pain, muscle pain, sight problems, disabilities, joint pain, body injury, loss of body parts, demotivation, organizational impact, mental stress, social impact, cardiovascular problems, respiratory problem, death, etc. are all dependent variables. On the other hand, independent variables are employment patterns (permanent, daily, and contract), Work experience, age, sex, residence, marital status, occupation, educational status. SEM need to select the variables as latent and measured variables so that variables are identified as stress, cardiovascular system, respiratory system, motivation, job satisfaction, and mental disorder are latent variables, on the other hand, temperature, humidity, injuries, death, working and rest duration, task load, age, work experience, educational level so on are all measurable variables.

#### 2.3.1 Research hypothesis and model development

The hypothesis and model have been developed based on previous studies and findings in different kinds of literature. The theoretical concept of the model is defined and described in detail in the previous chapter of the literature review portion of this paper. The causal relations and correlations of these variables in the model have been tested and estimated in the analysis part of the paper. The variable's direct and indirect effect and correlations are based on the last studies. Some direct causal relations or correlations will also not hypothesize. These two conflicts were corrected and seen in the analysis part. Causal relation model of occupational injuries and risk factors was modeled which the worker's occupational injury (endogenous dependent outcome variables) distribution might be brought from the independent and dependent exogenous variables. Rectangles show observed/measured variables, and ellipses denote for latent variables. Single-headed arrows show a directional effect, on the other hand, double-headed arrows show a correlation between two variables [20].

##### 2.3.1.1 Research hypothesis

As of Guo, Yiu, and González [21] Management safety commitment or working site factor was considered as one of the most fundamental safety climate factors for injury in any construction industries. Also this study concluded that safety related factors have a negative direct effect on the construction workers. Safety environment has also been observed by Bhattacharjee, Ghosh, and Young-Corbett [22] as a current-state consideration of the fundamental safety principles. Numerous socio demographic and work-related factors were included as control variables inducing both weekly working hours and injury [20]. This study founded that the mediating effects of gender, age, race/ethnicity, occupation, industry, type of pay, and psychological distress were simultaneously examined on the direct effect on body injury.

H1 Cumulative working site factors will have a direct significant impact on worker health & safety.

H2 Constructions safety risk factors will negatively affect worker's health and safety.

H3 Workers who are exposed to noise like, metal works (grinding and drilling, cutting so on) will have more chance of hearing problems.

H4 In socio-demographic factors, the worker having more experience and age will have less occupational injuries than workers having less experience and age.

H5 Worker's low salary income will directly increase worker's occupational injuries.

H6 The higher educational level will have less occupational injuries, which is the higher the educational level the lower the occupational injuries on the worker will be associated.

H7 In the constructions worker behavioral factors, job satisfaction will have negative direct effect (significant role on reducing occupational injuries) on occupational injuries.

H8 In the construction worker behavioral factors, personal protective equipment usage will have a negative direct effect on occupational injuries.

H9 Job stress level in workers behavioral factor will have a negative correlation with both personal protective equipment usage and job satisfaction.

H10 Working site-related factors will have a positive correlation with job stress but a negative correlation with job satisfaction and personal protective equipment.

H11 The safety factor will have a positive correlation with working site-related factors.

H12 In the construction worker behavioral factors, the job stress level will have a positive effect (significant role on increasing occupational injuries) on occupational injuries.

## 3 Findings

### 3.1 Descriptive statistics for the research variables

#### 3.1.1 Socio-demographic factor and characteristics of respondents

In this study from a total of 204 respondents majority of 66.2 % were male and the rest 33.8 % was female, also the majority of injury distribution was covered by males which were 57.1 % for males and 42.9 % were females. But it is seen that the value of the injured females were greater than not injured (16.9 %), the reverse is true for male which shows not injured in male was 83.1 %. All of illiterates, separated marital status, catholic, and other religious were injured all them, but the majority of the injury distribution has been covered by orthodox religious by 60.9 %, married (45.9 %) but less than not injured (50.7 %) workers and secondary school (23.3 %). Workers in a technique level have an injury level (10.5 %) greater than not injured (4.2 %) workers. However, the workers who have an educational status of master level were not injured all of them. In the case of monthly salary, most of the worker lays under a monthly salary of ETB (Ethiopian Birr) 1000–2000 which covers 27.9 % out 100 %. Also, the injury distribution shows 36.8 % which is greater than not injured workers (11.3 %). But the salary above ETB 5000 shows the injury distribution value was less than not injured values. Of all workers, 53.4 % of them work overtime (above 48), and 41.2 % work 48 h, and 5.4 % work up to 30 h per week. As it is shown, up to 30-h workers and above 48-h workers were much injured than 48-h workers. In the work experience, the result shows, as the work experience increases, the injury distribution decreases. If the overall injured percentage is less than the not injured percentage, in the sequence of increasing the work experience, it indicates the distribution of injury is decreasing. The experience having below 5 years were a more injured one. This result shows that the experience has a significant factor in reducing injury distribution.

#### 3.1.2 Behavioral factors of the respondents

**Fig. 3:**
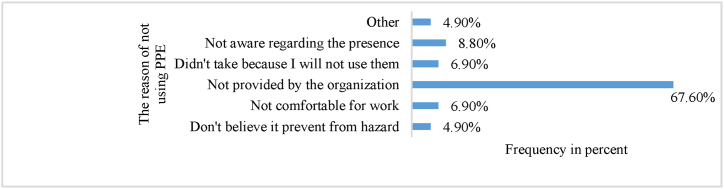
Frequency distribution of the reason of not using PPE in the site.

Above half percent of the respondents said that they were using a head mask (52.9 %) and wear a safety shoes (62.3 %), however, the rest of all protective equipment were not worn by above half percent of the worker in a day to day construction work activities. 94.6 % of the worker did not smoke cigarette, 61.8 % did not drink alcohol, and 81.9 % did not chow chat. In job satisfaction and stress level, 91.7 % of the building construction workers had a job stress case and 69.1 % of the worker was doing their tasks without satisfaction. Most of the construction workers didn't use PPE; the survey was designed for the reason of not using it. As depicted on Fig. 3, the reason was “not provided by the organization” takes the majority by 67.6 %, followed by “not aware regarding the presence” by 8.8 %, “not comfortable for work and didn't take because I will not use them” was equally 6.9 % and finally “do not believe it prevent from hazard and other reasons” equally takes 4.9 %.

#### 3.1.3 Working environment factors

**Fig. 4:**
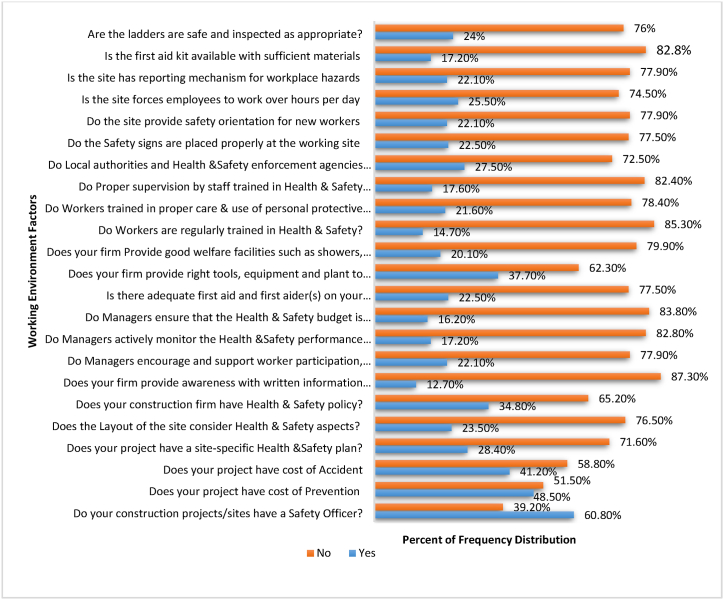
Frequency distribution of working site environment related factors.

The respondents were asked related to what the working site looks like, in case of safety and health considerations, general awareness on site-related work injuries and risk factors, in case of training, personal care, preventive costs& budgets, and other issues as shown on fig. 4. Of the total sites surveyed, 60.8 % or the response said that there was a construction site officer. This implies most construction sites had a safety officer. However, it was observed that the safety officer did not give safety awareness written documents, the response rate shows that 87.3 % said that awareness were not given, the rest 12.7 % said yes. The first aid kit were not even had a sufficient materials (82.8 %). They had a safety officer, but still, most building construction sites did not provide safety orientation for new workers.

#### 3.1.4 Safety risk factors

Of the risk factors those associates for occupational injuries, falling stairways, and ladders was covers a majority by 76 % and only 24 % of the worker did not fall at scaffolds and ladders. From those fallen workers 78.1 % was injured and 21.9 % was not injured, next was repetitive tasks by 70.1 %, from those repetitive task operators, 77.6 % were injured and 22.4 % were not injured. The third was hit by a fallen object with 68.6 % (75.7 % were injured and 24.3 % were not injured). Manual handling activities by 66.7 % (75.0 % injured and 25.0 % not injured), exposure to direct sunlight by 65.2 % (74.4 % injured and 25.6 % not injured), slip trip and fall by 64.2 % (77.9 % injured and 22.1 % not injured), also falls from height by 64.2 % (74.8 % injured and 25.2 % not injured) were the respective order of occupational risk factors. Although proximity to flammable or combustible materials by 42.6 % was the least injury factor and exposure hazardous substances with 43.6 % was the next least occupational injury factors. Of the total ladders, 84.8 % was made from wood, 9.8 % from metal, and 5.4 was from plastics. The injury distribution showed on the wood type of ladder by 67.6 %, which was 32.4 % not injured, next was metal by 55 % injured and 45 % not injured, and the last was from plastic which the injured (45.5 %) workers were less than injured (54.5 %) workers.

#### 3.1.5 Injuries (outcomes)

The overall injury distribution (prevalence) in large, medium and small scale building constructions for all body parts from all occupational risk factors associated on the building construction workers was 133(65.2 %) from a total of 204 responses taken.

**Fig. 5:**
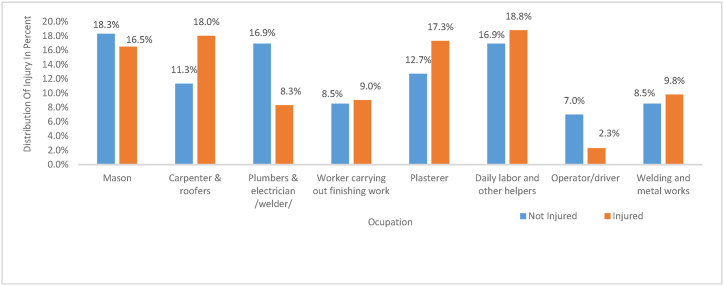
Workers working department versus overall injury distribution.

**Fig. 6:**
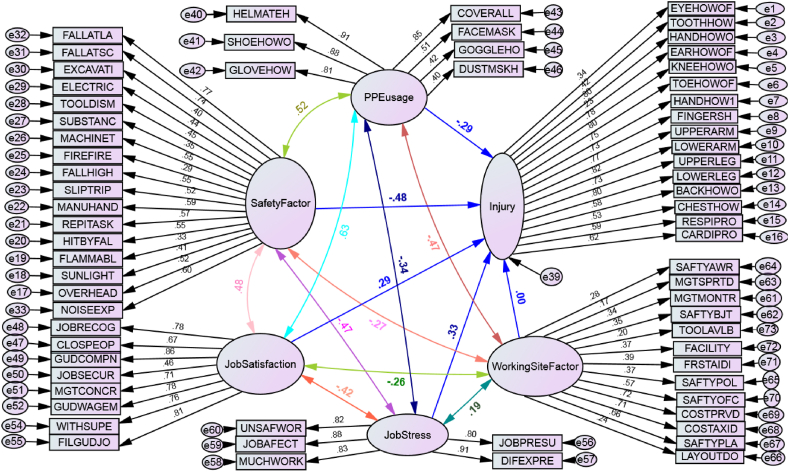
The SEM model estimation of each path.

Fig. 5shows, except mason, plumbers/electricians and operators/drivers, the rest occupations have injury distributions as injured individuals were greater than non-injured individuals. Workers, who carry out finishing work/painter/, are almost having equal injured and non-injured individuals. However, carpenter & roofers (18 %), plasterer (17.3 %), and daily labors and other helpers (18.8 %) were the most injury subjected occupations respect to others in respective order (see Fig. 6).

**Fig. 7:**
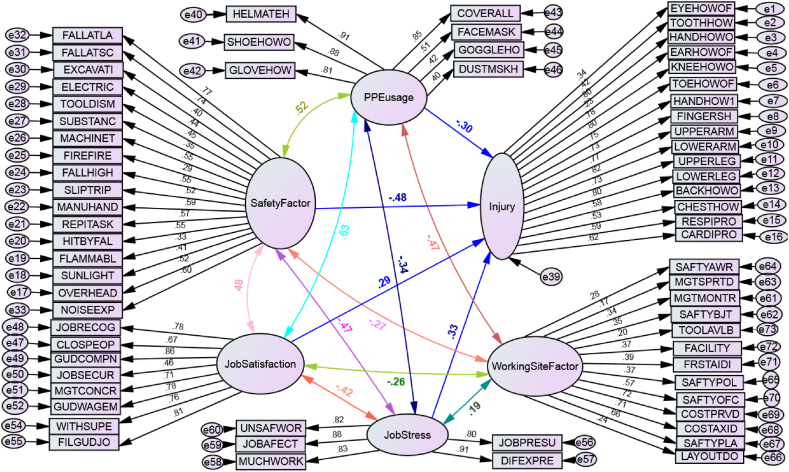
The modified SEM model.

Workers carrying out fishing works, daily labor/other helpers, and welding/metal workers were the only occupation mostly having eye injury. Also, daily labors were subjected to tooth and hand injury. Carpenters and roofers were suffering injuries mostly on hand, knee, toe, finger, hand, upper arm, lower arm, lower leg, and chest. But, of the total of 7.6 % for operator/driver and 18.1 % for welding and metal workers, all of them had an ear (hearing) problem. All of the respondents working on those two occupations were subjected to 100 % of hearing problems. Masons had the least injured body than the rest; it had only hand injury which had 18.2 % of injury coverage. Respondents were questioned like, when was the day of injury? At what time has the injury occurred? And what was your reason(s) at the time of injury? The majority 42.9 % of the respondents were injured Friday, followed by the worker do not remember the day of the injury occurrence, 15.8 % Thursday, and 15.0 % Saturday. From those, 57.9 % was injured afternoon, 18.0 % do not remember the time, and 15.8 % in the evening, because of 45.9 % it was due to not using PPE and followed by 15.0 % tiredness due to high work and 14.3 % was they think the accident was beyond control. This result implies workers were working till they were tired, because, as it is seen, the injury occurred afternoon and Friday.

### 3.2 Standardized Amos model estimation result

#### 3.2.1 Tests of model fit

Goodness of Fit Index (GFI) and Adjusted Goodness of fit (AGFI) was achieved, which was referred from Jöreskog and Sörbom [23], which was GFI less than 1 and greater than zero. The value near to 1 implies a perfect fit. The exogenous unobserved variables were the error terms with zero value. The factor loadings (path coefficients) values for unstandardized estimates have to be greater than 0.7; and also a value greater than 0.5 is too tolerable. Comparative fit index (CFI) that checks if the model fits data better than a model with no relationships among variables, the CFI must be greater than 0.731. Also, root means square error of approximation (RMSEA) which checks the average discrepancy between observed and predicted covariance and must be of less than 0.1 [24].

### 3.3 Interpretation of the model and hypothesis testing

This standardized regression weights shows; the safety factors were negatively affecting the workers body and behavior. There for H2 was **accepted**. When working Site factor goes up by 1 standard deviation, injury goes up by 0.005 standard deviations, when Job Stress goes up by 1 standard deviation, injury goes up by 0.326 standard deviations, which implies the direct positive effect(significant role on increasing occupational injuries), so that, **H12** was **accepted**. Job dissatisfaction was affecting the workers body as it was estimated. Therefore H7 Was **accepted**. When PPE usage goes up by 1 standard deviation and injury goes down by 0.294 standard deviations. This shows the negative effect on injury, therefore **H8** was **accepted**. However being the P value (0.942) was greater than 0.05, working site Factor had not a direct significant impact on construction workers that much. There for H1 was **rejected**.

On the correlation of variable the hypothesis were estimated as, working site related factors will have a positive correlation with job stress but negative correlation with job satisfaction and personal protective equipment (see Table 3). This hypothesis, H10 is **accepted**, because from Table 4 in the correlation on SEM path modeling, the correlation coefficient for work site factor and job stress was 0.19, for job satisfaction was −0.26 and for PPE usage was −0.47. Safety factor had a negative correlation with working site related factors. The correlation coefficient was −0.27, therefore H11 was **rejected**. Job stress level in workers behavioral factor had a negative correlation with both personal protective equipment usage and job satisfaction as it hypothesized the correlation coefficient were −0.42 for job satisfaction with job stress and −0.34 for PPE usage with job stress. Therefore H9 was **accepted**.

**Table 3:** Correlations: on SEM path modeling.

Exogenous latent variables	Bi-direction	Exogenous latent variables	Estimated value
PPE usage	<→	Job stress	−0.337
Safety factor	<→	Job stress	−0.474
Job Satisfaction	<→	Job stress	−0.416
PPE usage	<→	Job Satisfaction	0.629
Job stress	<→	Working site factor	0.189
Safety factor	<→	PPE usage	0.521
Job Satisfaction	<→	Working site actor	−0.257
PPE usage	<→	Job Satisfaction	−0.471
Safety factor	<→	Job Satisfaction	0.477
Safety factor	<→	Working site factor	−0.273

**Table 4:** Rank of the body mostly injured according to factor loadings.

Body parts	Causal direction	Exogenous latent variable	Factor load	Rank
Eye	<---	Injury	0.340	15
Cardiovascular	<---	Injury	0.623	10
Chest	<---	Injury	0.527	13
Respiratory	<---	Injury	0.593	11
Back	<---	Injury	0.576	12
LOWER Leg	<---	Injury	0.804	2
Upper Leg	<---	Injury	0.726	9
Lower Arm	<---	Injury	0.819	1
Upper Arm	<---	Injury	0.766	6
Fingers	<---	Injury	0.730	8
Head	<---	Injury	0.751	7
Toe	<---	Injury	0.797	4
Knee	<---	Injury	0.781	5
Ear	<---	Injury	0.233	16
Hand	<---	Injury	0.799	3
Tooth	<---	Injury	0.419	14

From the descriptive statistics of the research variables, the rest of hypothesis were tested. In the socio demographic factor, illiterates were injured them all, on the other hand the workers who have an educational status of master level were not injured all of them, this result has shown the educational level has a significance role on injury distribution, therefor H6 was **accepted**. Workers those who earn a monthly salary of above ETB 5000 shows the injury distribution value (percentage of injured workers) was less than not injured values, accordingly H5 was also **accepted.** In socio demographic factors, the worker having more experience and age had less occupational injuries than workers having less experience and age. As of the worker stays on the construction activities, the experience increases too, The experience having below 5 year were more injured than having the experience above 5 year. This result shows that the experience has a significant factor for reducing the injury distribution. So that H4 was **accepted**. Workers who are exposed to noise like welding/metal works and machine operators/drivers had also more chance of hearing problem. Of the total 7.6 % of operators and drivers, all of them were experience a hearing problem, also from the total 18.1 % of welders/metal workers, all of them were experience a hearing problem. Accordingly H3 was **accepted**. The injury distribution of building construction industries in Bahir Dar city was 133 (65.2 %). From those injury distribution, mostly affected body parts were selected out by using AMOS standardized model estimation. The significance level (P-value) of each injury was less than 0.05. The questionnaire for this survey was “was your cardiovascular system injured?” This question was also used for rest of injury variables. As it is seen from the ranking of the body parts injured, lower arm takes the majority of injury distribution by the estimated factor loading (path coefficient) of 0.819, this means, when lower arm goes up by 0.819 standard deviations, and injury goes up by 1 standard deviation. Next was lower leg by a Path coefficient of 0.804.

In H1**,** the cumulative working site factors had no a significant impact on worker health and safety directly. The significance level was greater than 0.05 which was 0.94. Therefore this estimated path is eliminated.

## 4 Discussion

The findings of this study have important implications for occupational health and safety in the construction industry. By identifying key ergonomic risk factors, this research provides a basis for developing targeted interventions to reduce injury rates. Additionally, the study highlights the need for policy changes to enforce ergonomic safety standards in construction projects.

The results indicated that 133 (65.2 %) of participants from a total 204 had injury during a building construction work in Bahir Dar city. It showed, more than half of the building construction workers had experienced occupational injuries of different types, which the proportion of occupational injury was much higher than what was reported in previous studies at different cities in Ethiopia. The prevalence reported by previous studies were, 38.7 % [7], 34.9 % [2], 46.2 % [8], 34.6 % [9], 41.4 % [10], 39.2 % [12], 32.6 % [38], and 43.9 % (Abate Lette, 2019) in Ethiopia and 76.2 % [11] and 66.7 % [13] which were out of Ethiopia.

The higher injury data observed in the present study could be attributed to several reasons. First, it could be related to the methodology used in this study, which considered structural equation modeling (SEM) instead of classical statistical tests in SPSS that optimizes the sample size. Thus, the sample size could have affected the accuracy of data. Second, it could be because of the study area, sample population experience requirement, and study season. Third, it could be because of the diversity of construction sites considered in the present study. Generally, previous similar studies have considered contractors of level one to level five, which have greater chance of employing safety and health related protocols than smaller contractors. It is possible that the combined small, medium, and large contractors considered in this study would have skewed the data.

Similarly, the distribution of injuries was studied for all body parts from all occupational risk factors associated on the building construction workers. The five primary frequently injured body parts were, lower arm with the estimated factor loading (path coefficient) of 0.819, lower leg (0.804), hand (0.799), toe (0.797), and knee (0.781). The rest respective order were, Upper Arm (0.766), Head (0.751), Fingers (0.730), Upper Leg (0.726), Cardiovascular (0.623), Respiratory (0.593), Back (0.576), Chest (0.527), Tooth (0.419), Eye(0.340) and the least body part that was subjected to the injury was ear. In the study of [25] also searched out the body injuries as neck pain 29 (7.5 %), shoulder pain 41(10.5 %), upper back pain 24(6.2 %), lower back pain 64 (16.5 %), legs pain 93 (23.9 %), and feet pain 52 (13.4 %). Also Boschman, van der Molen, Sluiter, and Frings-Dresen [26] supports the current study on the illnesses of the back, knee and shoulder/upper arm were the most prevalent among in both brick layers (masons) and supervisors, this direct effect on bodily injury, may be from other indirect effect of other main factors. Moreover[2] supports the current study, the primary injured body was hands (39.8 %), lower extremities (31.2 %) and upper extremities (arms and forearms). The most collective body parts injured were upper extremities by 31.0 % and lower extremities by 26.2 % in the study of Abbas et al. [8] and in Abate Lette ([34]) lower back (25.5 %), hands (16.9 %), and upper back (15.7 %) were the three primary types of bodies. In the study surveyed in Malaysia, the respective injured part were 45 % lower back, 28.3 % shoulder, 16.7 % neck, 15 % wrist or hand, 13.3 upper back, 13.3 % knee, 10 % elbow, 8.3 % hip or thigh, and 5 % ankle or feet [13]. The variations in different studies may be from country policies on the commitment of management, polices on working site, resource related phenomenon, contractor level selection in the sample, questionnaire design, sampling method, season of conducting the study, workers intensions while responding the survey and so on. The most frequently injury subjected occupations were, carpenter & roofers (18 %), plasterer (17.3 %) and daily laborer and other helpers (18.8 %) in respective order. This result has been supported by Chau et al. [27] and Khashaba, [41]El-Helaly, El-Gilany, Motawei, and Foda [1], thus studies found that, the injured workers were more likely to be carpenters, roofers and civil engineering workers were more exposed one. For the current study operators/drivers had the least frequency of injury occurrence. Like the current study, drivers and crane operators had also the last injured occupation in Aneziris, Topali, and Papazoglou [28]. Majority of the injuries were occurred on Friday (38.2 %) and afternoon time (50.0 %) working on above 48 h (53.4 %), do not wear PPE (37.7 %) because of not provided by the organization (67.6 %). This result implies that at the end of the week, end of the day and working overtime; a cumulative work load were affecting to be tired which could lead them un-capable of lifting an object and perform the task, could not protect themselves which caused them to be injured. Being they were not wear PPE injury will be hurt them. In the current study; the distributions of injuries over the workers physical and mental dynamics, were due to different occupational risk factors. The significant factors for those injuries were, PPE usage, job satisfaction, drink alcohol, chow chat, gender (Being female), climbing ladder, carry tool while on ladder, climb or work on any scaffold, do not tie belt at scaffold, carry heavy or awkward objects long, distance, wage, layout didn't consider health & safety aspects, not provide safety orientation for new workers and all of 17 safety risk factors. But the most leading factors of occupational injuries were selected. The six most leading and prioritized risk factor of occupational injuries were, do not tie belt at scaffold, falling stairways and ladders, exposure hazardous substances, tools and machinery, electricity (electric power accidents) repetitive tasks the layout didn't consider health & safety aspects in the site and do not provide safety orientation for new workers engaging the job. Nguyen and Tran [29] had also reported that falls from height were leading cause of fatalities and serious injuries in construction. Also, [8] the common source of injuries found were falls (47.6 %) and harms by manual tools (23.8 %) were the main causes of injuries. Those results (falling) implying that, the workers climbing a ladder, scaffolding and stairways, were not tying the belt, maybe that's why the two consecutive risk factors were a found to be in sequence. In the confirmatory factor analysis (hypothesis testing), construction site related factor was estimated as, it would significantly affect the workers' health and safety, but it was not directly generating the injury, however it was indirectly affecting the worker.

## 5 Work practices recommended to construction sites

National institute for occupational safety and health (NIOSH), the national treasury of republic of South Africa, and other studies has conducted different recommended construction practices that had been reduce occupational safety and health injuries in construction industries. For the better work environment, better safety and comfort, and minimum occupational injuries, the following work practices that has been taken from different studies tested in practical are recommended. Those studies were ([30,31]; Ayat Al swaity, [36]; P. Entzel, J. Albers, & L. Welch, [44]; [32,33]). The construction contractors, owners, workers, government bodies, supervisors and safety experts in the construction site is better together to implement those simple solutions and practices given below for each work area. Those work practices are given prior to the more significant risk factors found in our study result. In floor and surface work that requires stooping, bending, twisting, kneeling or squatting for long period, workers are devised to use screw guns and other fastening tools, auto feed screw gun with an extension which allows the worker to stand upright while working, use a powder-actuated fastening tool, use motorized concrete screeds, use rebar tying tool (for workers tie the rebar by their hand with pliers and tying wires even without having glove and safety shoes), use kneeling creepers (the floor having hard surface hurts workers knee and arm as well by directly applying the pressure on ligaments, tendons and ligaments of the knee joint which lead serious back pain and knee and arm gangrene), and use flexible scaffold for masonry activity. For overhead work which has to be done above the shoulder level of one hand or two hands, the head of the worker leans up and tilted back to see the work for drilling, cutting, welding, fastening, and fastening and so on. For such work activity, Use bit extension shafts for drills and screw guns, Use extension rods for powder-actuated tools, and Use pneumatic drywall finishing systems. For lifting, holding, and handling materials, The methods that will reduce such risk is, change the materials or working process (use alternative tools and components or work methods those are less labor-intensive which can be handled without requiring high physical effort, requiring awkward position, and which not need frequent motion), change tools or equipment (the owners who have no capable of buying it can even take a rent. Special round tools or materials which their edge are not sharp is recommended, materials comfort for handle and easy to carry is best preferable, hydraulic and vacuum lifting materials which can lift heavy to workers in the construction, especially for windows work and sheet metals), change work rule (owners/contractors/managers can make policies of storing and release it from store which requires at a height with respect to workers capable to reach it easily, and also layout of materials in the store is better think the principle of first in first out, first in last out, last in last out and last in first out policies.) and provide training (in case of how the tool should be handled the worker, especially new workers is the better solution for reducing risk, this method is not only for handling purpose, but also for any safety purpose, training is the key solution reducing injuries and improving productivity. Example the method how the material lifts is using two handed grip by kipping the back straight which should not exceed ten inches away from the body). For the workers perform their task by gripping tools by hands in full time of the shift; use ergonomic hand tools, use pinching, riveting, or cutting tools (instead of try to open and cut materials in hands), use anti-vibration gloves and shoulder pads.

## 6 Conclusion

The results of this study revealed that out of total participants 65.2 % of the workers experienced occupational injuries which was much prevalent than most of the studies conducted in some parts of Ethiopia. The five primary frequently injured body parts were; lower arm with the estimated factor loading of 0.819, lower leg (0.804), hand (0.799), toe (0.797), and knee (0.781). The most frequently injury subjected occupations were, carpenter & roofers (18 %), plasterer (17.3 %) and daily laborer & other helpers (18.8 %) in respective order. The most significant factors for injuries were caused by not tying belt at scaffold, falling stairways and ladders, exposure to hazardous substances, tools and machinery, electric power accidents, repetitive tasks, poor layout that didn't consider health & safety aspects in the site and by not providing safety orientation for new workers. Therefore, implementation of basic occupational health and safety services in building sites including providing awareness, facilities, protective measures, supportive management commitments, selecting appropriate machines/tools, reducing repetitive tasks, improving layout, maintaining regular inspections on scaffolding/ladders and substituting wood scaffoldings with metals or plastics are highly advisable to reduce the overall injury distributions over human body dynamics, mental aspects, and social loses.

## Future studies

Since limited ergonomics related researches are conducted, especially in the construction sector, it is difficult to improve workplaces and create a good balance between human capabilities and the day to day activities. Therefore, researchers are advised to put their effort on such area by implementing ergonomics and safety principles and guidelines. In the present study, because only some building constructions were registered and are reporting accidents to the labor and social affairs and administration bureau, fatal and death accidents might not have been fully addressed. Also, because of the lack of documentation and unorganized recording system in labor and social affairs and administration bureau, it was difficult to select out fatal accidents and deaths’. The workers having near or more than one year could have biased data depending on how much they had remembered, which might lead to some over or under-reporting of responses. Therefore, future studies are required to consider larger sample size and further analysis of measures to improve the prevalence based on the prioritized factors.

## CRediT authorship contribution statement

**Gebeyaw T. Belay:** Writing – review & editing, Writing – original draft, Visualization, Validation, Software, Methodology, Investigation, Formal analysis, Data curation, Conceptualization. **Bereket H. Woldegiworgis:** Supervision. **Yogi T. Prasetyo:** Software.

## Declaration of competing interest

The authors declare that there is no conflict of interest.
